# Δ 9-Tetrahydrocannabinol Toxicity and Validation of Cannabidiol on Brain Dopamine Levels: An Assessment on Cannabis Duplicity

**DOI:** 10.1007/s13659-020-00263-z

**Published:** 2020-08-28

**Authors:** Swapnali Chetia, Gaurab Borah

**Affiliations:** Department of Zoology, Rajiv Gandhi University, Rono Hills, Doimukh, Arunachal Pradesh 791112 India

**Keywords:** *Cannabis*, Tetrahydrocannabinol, Cannabidiol, Dopamine, Medicinal uses

## Abstract

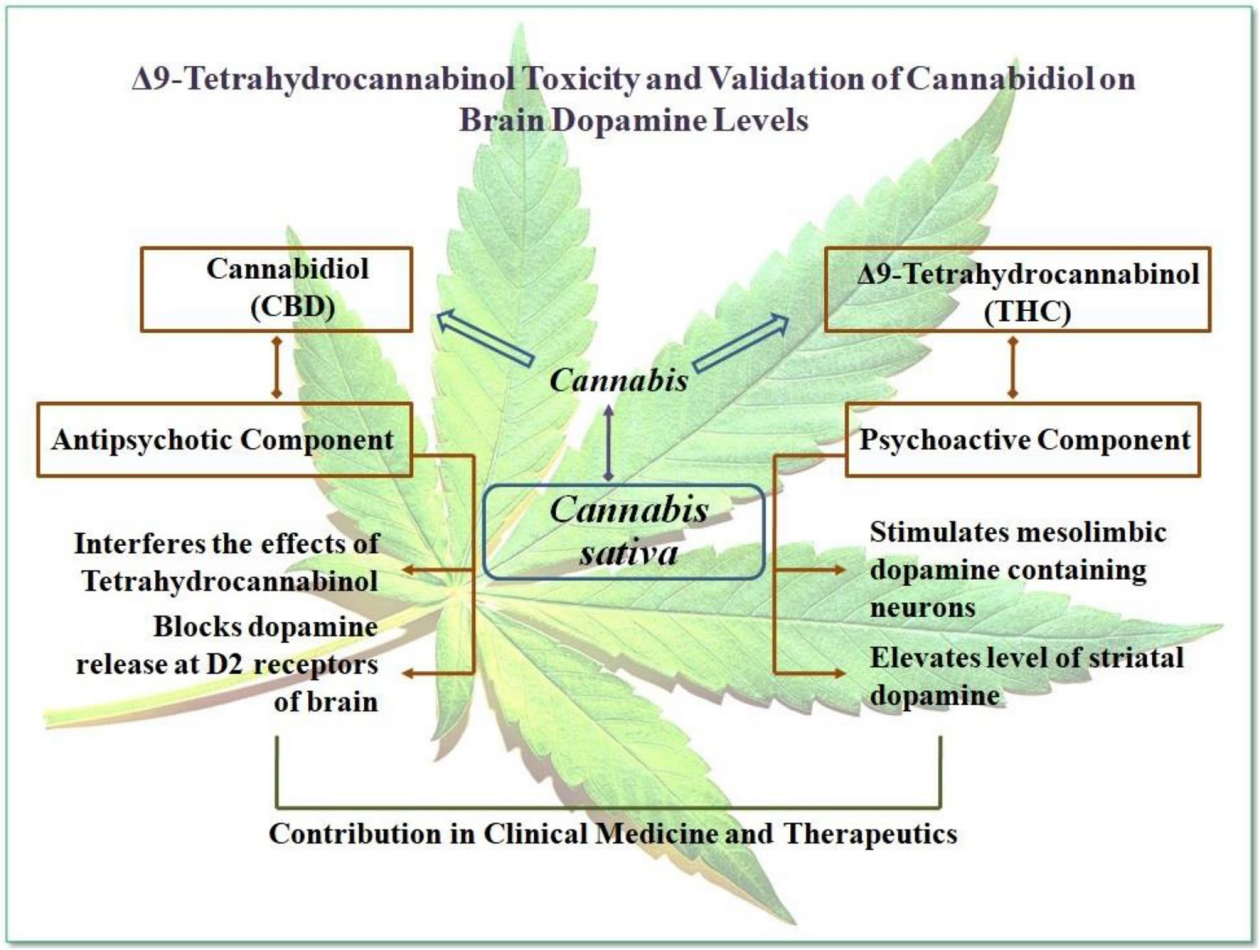

## Introduction

Cannabis, commonly called marijuana, is a psychoactive and one of the most illicit recreational drugs around the world [1, 2], extracted from *Cannabis sativa*. With a precise estimation, as reported in 2017, there are about 188 million cannabis users in the world, rating the world’s population at approximately 3.8% [3]. Cannabis possesses antioxidant properties which may also contribute to other therapeutic aspects like anticonvulsant, anti-inflammatory, and neuroprotection, other than its detrimental effects [4]. Acute and chronic use of cannabis can influence CNS and PNS through several complications which include hyperemesis syndrome, impaired coordination, and performance, anxiety, psychotic disorders, suicidal tendencies, cannabis withdrawal symptoms, neurocognitive impairment, cardiovascular, respiratory, Cerebro-peripheral vascular diseases [1, 5], bullous lung disease, pneumothorax, desquamated interstitial disease, pneumopericardium, pneumomediastinum, and brown pigmented macrophages [6].

**Fig. 1:**
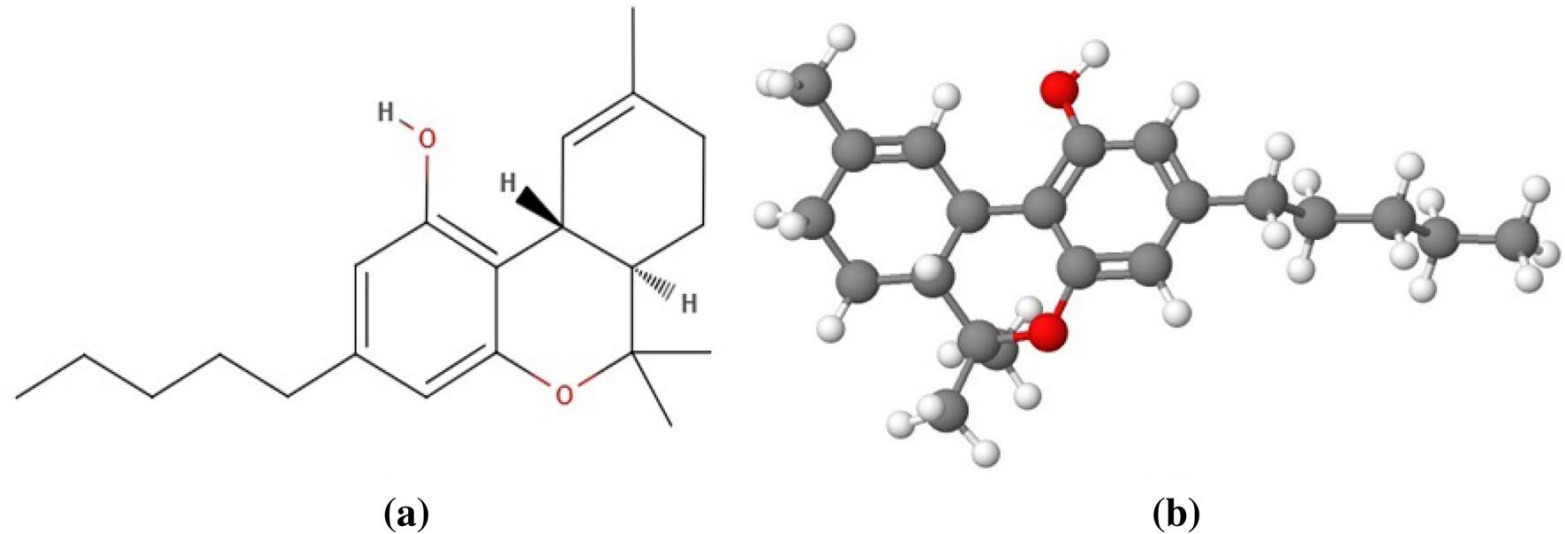
Structural representation of Δ9-tetrahydrocannabinol **a** structural arrangement **b** 3D- model of the structure

**Fig. 2:**
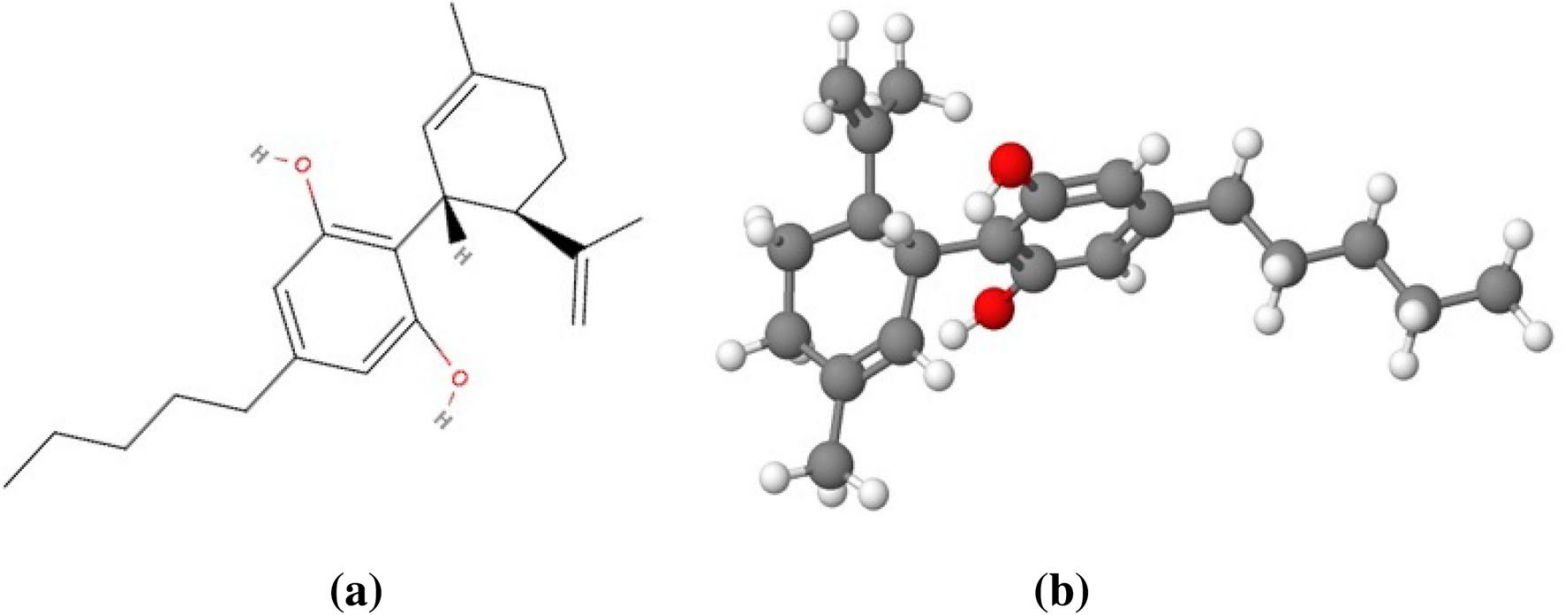
Structural representation of cannabidiol **a** structural arrangement **b** 3D- model of the structure

Apart from the virtue of medicinal properties, recent studies on chronic cannabis inhalation reported the intimation of cerebrovascular diseases [7] although the underlying mechanisms have not been strongly established yet. Numerous neurological disorders have been observed in discrete studies on cannabis exposure viz. cognitive dysfunction, behavioral complications, memory/attention deficiency, structural and functional variations in the brain [8–11]. About 100 cannabinoids are being reported to date [12], out of which Δ9- tetrahydrocannabinol (THC) (Fig. 1) and cannabidiol (CBD) (Fig. 2) are the principal components that are actively involved in brain DAergic alterations. THC is one of the major and principal psychoactive constituents of cannabis [13]. In contrast, CBD is an anxiolytic and antipsychotic cannabinoid compound that may help in inhibiting the effects of THC and other negative effects effectuated due to cannabis exposure [14–16]. The psychoactive properties of THC are responsible for cannabis addictive potential and alterations in brain dopaminergic (DAergic) functions. Acute THC administration has been reported to elicit striatal dopamine (DA) release in animals [17] and humans [18–20].

### Cannabis Addiction

**Fig. 3:**
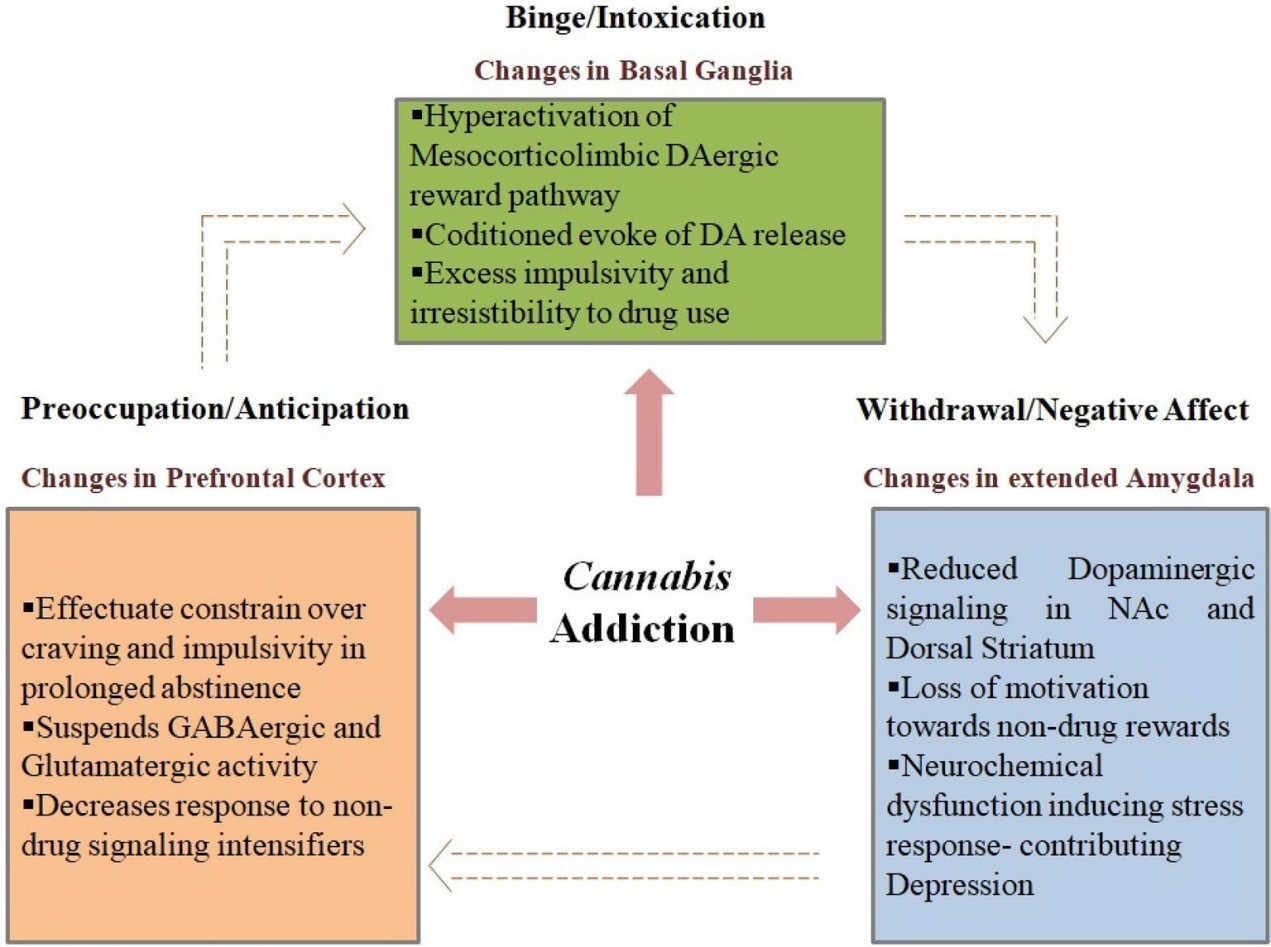
Stages of drug addiction: binge/intoxication stage, withdrawal/negative affect stage and preoccupation/anticipation stage

Drug or cannabis addiction is a compulsive drug intake disorder, embodied due to loss of control over drug intake limitation and when prevented, emerges a negative emotional state [21]. THC is the principal causative cannabinoid responsible for cannabis addiction due to its psychoactive properties and associated effects on brain DAergic function. Koob and Volkow [21] described three stages of drug addiction that confer major alterations in neurocircuits. The three stages are—(a) the binge/intoxication stage generated by changes in basal ganglia characterized by excess impulsivity and irresistibility to drug usage despite the detrimental effects associated with it, (b) the withdrawal/negative affect stage driven by changes in the extended amygdala involving stria terminalis that implies reward cognition due to the loss of motivations towards non-drug rewards, (c) the preoccupation/anticipation stage driven by alterations in the prefrontal cortex (PFC) involving disrupted GABAergic and glutamatergic activity (Fig. 3).

In the intoxication stage, the mesocorticolimbic DAergic reward pathway of the brain is hyperactivated followed by positive reinforcement of the rewarding effects of drugs. Impairment in incentive salience is a characteristic feature of the intoxication stage in which initial exposure to a drug intimating signaling contexts are exaggeratedly assigned with high rewarding properties. This lead to a conditioned evoke of DA release. Incentive salience dysfunction drives DA signals to perpetuate impulse of the drug upon exposure to conditioned-cues.

Following the intoxication stage, the withdrawal stage is triggered by adversary process responses following an overdosing interlude. These adversary process retaliations are marked by neurobiological within and between-system changes that direct motivation loss towards non-drug rewards and impaired emotion regulation. Within-system neuroconversions encompass dorsal striatum and the nucleus accumbens (NAc) with reduced DAergic signaling, resulting in elevation of reward thresholds for non-drug promoters, promoting depression. Between-system neuroadaptations embrace neurochemical dysfunction induced stress responses such as elevated extrication of corticotrophin-releasing factor (CRF) in the amygdala inclusive of HPA-axis dysfunction. These reverberates distinguishing symptoms specifically chronic irritability, anxiety-like responses, malaise, and dysphoria.

The preoccupation stage is involved in rehabilitation-induced abusive substance ensuing abstention. In this prolonged abstinence, effectuated constrain over craving and impulsivity is a vital strategy interposed by the PFC. This stage is marked by self-regulation, decision making, inhibitory control, and functional memory influenced through aberrant signaling between PFC and brain. This in turn might suspend the activity of GABAergic and glutamatergic exploitation. Proprietarily, this renders surplus salience incrimination of drug-induced cues, reduction in responsiveness-induced non-drug signaling intensifiers, and reduces maladaptive behavior inhibition activity.

## Cannabis and Dopamine

Vulnerability to DA-enhancing substances of abuse is analogous to impaired striatal DA transmission [22, 23]. Studies in chronic cannabis users (CD) with co-morbid psychotic symptoms unveiled a reduction in DA synthesis [24] and mitigate the release of stress-induced DA [25]. This exhibits an inconsistent effect of reduced DA release in chronic cannabis users without psychotic symptoms. These inconsistent effects are relevant to concomitant use of cannabis along with other non-specified drugs, which depends on the cannabis severity. Through positron emission tomography (PET) [11C] raclopride, it is found that severity of cannabis use vary across cohort limitations of DA quantification changes. It was earlier demonstrated that the use of cannabis induces a deficit in neurocognitive function [26] and decreases striatal DA action [27, 28]. It was also predicted that in chronic cannabis users, reduction in DA release would counter-correlate with neurocognitive function and effectively assign psychosis-related symptoms [29].

It has been reported that upregulation of glutamate levels in the striatum and hippocampus could lead to schizophrenia [30, 31] and the use of cannabis induces psychosis [32]. In a cohort, evaluation of striatal-hippocampal glutamate levels and striatal DA release were juxtaposed through magnetic resonance spectroscopy (MRS) [29]. They highlighted that cannabis could alter glutamate levels in the hippocampus and striatum along with alterations in DA release. Thereby, reduced striatal DA release could promote psychopathology in cannabis users.

Although, it was demonstrated that, in cannabis users, reduced striatal DA release affects associative striatum (AST) and sensorimotor striatum (SMST) regions in contrast to other drugs that induce limbic striatum (LST) DA release deficit and effectuate intense seeking/craving [22, 23]. A possible reason could be the distribution of distinctive anatomical targets of THC, CB1 receptor. As reported, CB1 receptors are rarely present in the ventral striatum in contrast to the putamen or dorsal striatum in humans [33] or rat brain respectively [34, 35]. Besides, it was also documented that, in humans, numerous CB1 receptors are present in the pallidus as compared to thalamus [36]. Reduction in striatal DA release, however, is relatable to a tedious performance in working memory [26]. Conversely, higher striatal DA release engenders intense performance of the same [27, 28]. It is eminently suggested that cannabis use in adolescence may promote drug dependence associated with a compromised DAergic system that may alter brain function.

### Δ9-Tetrahydrocannabinol (THC) and Dopamine

Δ9-tetrahydrocannabinol (THC) of cannabis is the principal psychoactive constituent that expends its effect on the brain. THC is an endocannabinoid receptor (CB1R) and CB2 receptor (CB2R) partial agonist, which is extensively embodied in the basal ganglia and SN pars reticulata of the brain [36, 37]. THC stimulates mesolimbic DA containing neurons and elevates the level of striatal DA in animals [38]. In some studies, in humans, acute THC is reported to instigate striatal DA release [19, 39, 40], but reports also exist that don't support this statement [41, 42].

CB1R antagonist rimonabant, through the activation of G-protein-coupled CB1R receptors, blocks the execution of psychoactive effects of THC [43, 44]. This mitigates the levels of cAMP by retarding the expression of adenylate cyclase [45]. THC disrupts the calibration of CB retrograde signaling systems on account of both its temporal and neuronal specificity over THC. Under conditions of a low density of CB1R, THC aggravates endogenous agonists procuring greater receptor efficacy than THC [46]. Through the allosteric activity, THC modulates opioid receptors [47], which may additionally dispense indirect routes for variations in DA transmission [48].

#### CB1 Receptors and Dopamine

Earlier, studies in animal models, demonstrated that amphetamine (AMPH) increases DA release when interacted with THC [49]. These reported that depending on the dose of THC, the behavioral effects of amphetamine may vary [50]. Indeed, contributing to the recreational and detrimental effects of cannabis, involuted THC generates complications on the DA system.

It is reported that the endocannabinoid complex modulates the functioning of the DAergic neuronal system [51]. Anandamide and 2-arachidonoylglycerol (2-AG) (endocannabinoid ligands) along with CB1Rs are abundantly present in the DAergic pathways [52]. Inevitably, they voluntarily exploit as a retrograde feedback system to regulate DA transmission on presynaptic GABAergic nerve terminals. These endocannabinoid ligands, consequently, vitalize the release of DA in the NAc shell [53]. CB1 antagonist rimonabant, stipulating endocannabinoids induced DAergic effects involving CB1R, blocks the modulation of this process. THC reward over increased DA release and DAergic neuronal discharge are reinforced by partial signal transduction mechanisms from CB1R [43].

#### Studies in Acute THC and Dopamine

In vitro radiolabelled studies of DA in synaptosomes of rodents revealed that THC induces increased DA synthesis [54] and release [50]. However, the effects of THC on DA activity laid a conflict with the evidence of both its elevation [55] and dose-dependent reduction [50]. Subsequently, it was reported that a lower dose of THC increases throughout the process of conversion from tyrosine to DA, whereas high doses resulted in decreased DA synthesis [56]. However, monitoring the correlation of THC administration and levels of DA alterations executed that repeated dosing of cannabis may result in behavioral and neurochemical fluctuations which may relevantly provoke drug dependence. In rodents, due to reduced precursor uptake [55] and μ-opioid receptors-mediated DA-opioid reciprocity, reverberation of dose-specific THC is assumed to occur [54].

In an in vivo study, THC is found to aggravate the rate-limiting strategy of DA synthesis pathway by increasing [3H]-DA synthesis [57, 58], tyrosine hydroxylase [59] and mRNA expression [60]. Similarly, in most rodent studies, demarcation with 3,4- Dihydroxyphenylacetic acid (DOPAC)/DA ratio also exhibited increased DA metabolism [61] despite other contradictory studies [59].

Contrary to the above findings, Acute THC, in rhesus monkeys, did not alter the protein levels in the DA receptor [62]. One study in the limbic forebrain of rats reported increased availability of DA type 1 receptor (D1R) [63] in contrast to other studies that showed decreased accessibility [64]. While, in the striatum, decreased or null significant changes in the density of DA type 2 receptor (D2R) was reported [64] with a decrease in D1R [63]. A study on human brain activity through functional magnetic resonance imaging (fMRI) and PET demonstrated that DAergic activity may alter due to the changes in glucose metabolism and cerebral blood blow. These serve as a substitute marker in areas with DAergic prognosis activity of the brain. Due to direct endocannabinoid effects, acute THC and high concentration of CB1R intensifies the activities in frontal and subcortical regions of the brain in humans [65, 66]. Through a study in the striatum of a resting brain containing dense DAergic innervations, inconsistent effects were found pertaining to both elevated and reduced activity of acute THC [65]. However, DAergic signaling modulates certain cognitive ventures which may provide a vigorous substitution for DAergic transmission induced by THC. For instance, inhibition of motor response is accompanied by cortical DA release. Thereby, THC is found to attenuate its activity in the inferior frontal cortex (right) and anterior cingulated cortex (ACC) in humans previously exposed to cannabis [67]. A moderate DAergic activity was made evident in healthy humans previously exposed to cannabis; whereby THC induced striatal activation impairment [68]. In contrast, a study in occasional cannabis users, THC was found to induce a predominant impairment of brain responses in commentary reward trials [69]. Molecular imaging on the effect of acute THC on the DA system in humans with previous exposure to cannabis also revealed that THC stimulates DA release in the ventral striatum of the human brain [19].

### Cannabidiol (CBD) and Dopamine

The first study on CBD induced anxiolytic and antipsychotic effects was demonstrated in the 1970s and 1980s and was later studied in humans executing promising results [70]. In addition to anxiety and psychotic effects, basic and clinical research on other therapeutic contributions of CBD was conducted. Moreover, synthetic analogs of CBD with efficacious potentiality have recently been developed recommended for patients with compromised health [71]. CBD was first isolated from cannabis extracts by Adams and his co-workers in the year 1940 [72]. In the early 1970s, several studies reported that CBD was incompetent to imitate the effects of cannabis, which led to hypothesize that; CBD would be a non-functioning cannabinoid. However, it was also speculated that CBD, with other cannabinoids, could interfere with the THC effects [73, 74]. These studies indicated the pharmacological activities of CBD exhibiting a broad spectrum of gesture [70].

Although, it is believed that CBD acts on DA receptors to induce its effects, there are no known reports that manifested its direct effect on D2 receptors of DA. Depending on this statement, a group of researchers recommended that CBD is the principal superficial exception in blocking or interfering DA at the D2 receptors of the brain DA system [75, 76]. Previously, it was reported that the antipsychotic effects of CBD did not attribute to DAergic regions of the brain or any other brain receptor [77, 78]. Although it is a known fact that DA D2 receptors are the main target for antipsychotic drugs, it has been a prime factor of research on CBD to prove its antipsychotic property on D2 receptors of DA [76]. Experimental evidence helped to understand some of the effects of CBD that acts as an atypical antipsychotic drug [79–81]. Δ9-THC acts as a partial agonist at the G-protein-linked receptor at the rat cerebral cannabinoid, whereas CBD behaved as an antagonist at a range of micromolar concentrations [82].

Recent evidence suggested the antipsychotic effect of CBD as a promising potential antipsychotic treatment. In preclinical models of schizophrenia induced by psychometric drugs, CBD is found to reduce its detrimental effects by its neuropharmacological profile. As mentioned, CBD is found to be more effective than haloperidol and analogous to clozapine in attenuating ketamine-induced hyperlocomotion [80]. In experimental mice and rats, CBD has been reported to converse MK-801-induced sensorimotor gating deficits [83] and social withdrawal respectively [84]. In 2012, Leweke et al., documented in a recent clinical trial that, CBD possesses antipsychotic properties which reduce psychotic symptoms with significantly fewer side-effects [78]. However, the mechanisms underlying the antipsychotic effect of CBD is still unknown. Considerably, molecular evidence on schizophrenia explains disturbances in signaling pathways along with DA receptor function. These signaling pathways include the Wnt signal transduction pathway, Akt, GSK-3, and catenin. Most eminently, both typical and atypical medicaments can initiate these pathways [85–87].

Again, in a contemporary study, CBD is reported to attenuate AMPH-induced psychomotor sensitization and sensorimotor gating deficits [88]. They also intimated that CBD generates its effects through the modulation of mTOR/p70S6K signaling pathway phosphorylation in the NAc shells. Furthermore, CBD is documented to accustom the dysregulation of mesolimbic DA neuron activity states induced by AMPH. They also reported that CBD blocks modulation of mesolimbic DA and dysregulation of striatal activation patterns induced by acute THC. Corresponding to these findings, Englund et al. reported that pretreatment with CBD is potent against antipsychotic effects when assigned prior to the administration of THC [89]. However, Renard et al. [88] demonstrated a novel mechanism on the putative antipsychotic effects of CBD with the mesolimbic system. They justified their demonstration through animal models validated with DAergic sensitization. They reported CBD to attenuate AMPH-induced sensitization and activity of DAergic neurons within the ventral tegmental area (VTA) in the NAc shell. Additionally, they reported that CBD was dependent on the mTOR/p70S6K signaling pathway elevating its phosphorylation state and blocking these molecular effects in the NAc shell. It culminates that CBD directly acts on the mesolimbic pathway and DAergic functions which is a critical underlying variable in cannabis-induced psychometric complications [90, 91]. mTOR signaling is an analytical modulator of synaptic plasticity, neuronal morphology, and functional memory [92–94] and has been a growing interest of research over the past decades.

Preclinical studies accounted for a significant reduction in mTOR/p70S6K signaling in the PFC of patients experiencing depression [94], where, ketamine (noncompetitive NMDA receptor antagonist) imply rapid anti-depressant effect by modulating mTOR activation [95]. With the consistent findings, it can be disclosed that CBD as an antipsychotic medication can directly act upon DA D2 receptors of the striatum by increasing mTOR/p70S6K signaling. As per the mechanism of CBD is concerned, the VTA and NAc equate a common association via DAergic and GABAergic afferents from the VTA and GABAergic efferents from subpopulations of NAc. The medium spiny neurons that evolved protrude back to the VTA [96–101]. The GABAergic projection is anticipated to arbitrate a “long-loop” inhibitory feedback to regulate VTA DA neurons [101–103].

## Cannabis in Medicinal Implementation

In spite of the detrimental effects associated with cannabis use, the medical implications of the plant (*Cannabis sativa*) have gained much interest during the last 20 years [104]. The use of cannabis (marijuana) was accepted and validated in 1999 by the National Academies of Sciences, Engineering, and Medicine; and accordingly, managerial medical colleges recommended for its prescription to patients [105]. In 2017, an updated report called for a national research agenda, enhancement of research quality, up-gradation of collected data with surveillance endeavors, and strategies for an inscription of barriers in advancing the schedule of cannabis [106].

Over the last decade, in North America, there has been an increased interest in the use of medical cannabis. It is estimated that about 3.5 million people in the USA are purposely using medical cannabis legally, and is recorded for a total worth of USD $ 6.7 billion are endowed in North America on legal marijuana in 2016 [107]. Health care of Canada approved the Canadian residents to purchase medical marijuana with prescriptions, which boosted its use from 30,537 in 2015 to near 100,000 in 2016 [108]. Medical cannabis assists the use of its components in the varied extent of medical conditions, remarkably in the field of pain management [109] and multiple sclerosis [110]. Numerous synthetic cannabinoids are useful in medicinal purposes and are already been produced viz. dronabinol and nabilone, which, in some ways, mimic the effects of THC. They are licensed by many countries including the US, Netherland, Germany, Austria for the treatment of weight loss in patients suffering from nausea, vomiting, and AIDS [16]. In comparison to other herbal remedies, the prepared content of non-medicinal CBD, as declared, is often inaccurate [111, 112], and these products periodically may exceed the legal limit of THC [112]. Moreover, when compared to the clinical trials [113], the amount of CBD in these products is found to be much lower [114, 115]. There are some products composed of cannabis that were already available in the medicinal ground before its rescheduling in 2018. Sativex, derived from cannabis, is an oral spray that contains both THC and CBD in a 1:1 ratio. It is licensed in 29 countries including Canada and the UK and is used for the treatment of multiple sclerosis spasms. However, a meta-analysis suggested that its effectiveness may be inadequate and restricted [116]. Because of its weak and insufficient effectiveness, it is not recommended by the UK's National Institute for Health and Care Excellence (NICE) [117]. Additionally, a cannabis-derived oral solution named Epidiolex was licensed by the US Food and Drug Administration in 2018 for seizure medicaments in two rare and severe forms of childhood epilepsy—Lennox–Gaustat syndrome and Dravet syndrome [113].

Products of plant-derived cannabis are not well evaluated on the basis of traditional medicines, which led to an increase in uncertainty regarding its aptitude in human health [109]. While, it is well understood from these findings that there are many synthetic forms of cannabis that are easily accessible with prescriptions, but the various cannabis plant products obtained naturally differ in the concentrations of THC and CBD which makes the effects of exposure unpredictable [118].

## Discussion

Cannabis is a known illicit drug that has been used for many decades. There are above 100 cannabinoids reported to be present, out of which THC is the causative incompetent cannabinoid that causes DA insult; whereas, CBD is a positively featured cannabinoid with antipsychotic effect. Behind these findings, the presence of other cannabinoids of cannabis is not yet isolated or introduced that could explain their possible effects on brain DA manifestations. This review highlights the evidence showing that THC exerts its detrimental effects on the DA system [19, 39, 40, 48]. The various research work and studies demonstrated that acute administration of THC elevated DA release and nerve activity which are region-specific and understanding their functional significance is the basic need. The available preclinical evidence suggested that the administration of chronic THC induces long-term complications on the DA system. There is a need for further studies on the use of cannabis in association with other drugs like nicotine and alcohol when administered together. There is also a need to understand the activities of other cannabinoids of *Cannabis sativa*, their role in the moderation of THC-induced DAergic alterations. Human PET studies have executed reports on blunted DA synthesis in cannabis users in contrast to non-users, the precise mechanisms underlying this process is not yet clear.

Besides the knowledge on the increasing use of medical cannabis in most countries, an understanding of the landscape of available evidence syntheses is needed to support results across reviews, including non-synthesized (study-by-study) data [107]. Many studies were unable to furnish a definitive statement regarding the positive repercussions of cannabis viz. pain management and multiple sclerosis [104]. Much research has been carried out on the hazardous effects of cannabis, providing relevance on THC menace, its jeopardy in DA synthesis and release, its effect in the DA receptors as well as NAc in regular cannabis users. Even so, the antipsychotic effect of CBD is not well evaluated in the DAergic region of the brain, which can elaborately explain its efficacy in DA release and its’ relation with DA receptors.

## Conclusion and Future Perspectives

Individuals may be influenced by an addiction arrest through various biological and sociological factors. This may induce vulnerability towards initial use in addition to the positive and negative reinforcement that follows. This vulnerability is further complicated with a comorbid psychiatric disorder and withdrawal of the same is followed by self-medication. The evidence highlighted in this study revealed the subversive effects of recreational cannabis rather than therapeutics. Evaluation and analysis of related findings is a measure to disseminate accurate information to the people so that individuals can resolve and make precocious choices on their use.

Precisely, acute exposure to both natural and synthetic cannabinoids engenders a complete array of transitory symptoms, cognitive deficits, and psychophysiological abnormalities. Eventually, cannabis exposure in adolescence yields a higher risk of psychosis, although dose-dependent. These relevant findings may contribute caution on cannabis-induced health criticism including global and specific domains of cognitive impairment which may be irreversible. Additional research and standard epidemiological studies are needed to further specify the extent of the adverse effects caused due to cannabis use.

Through these findings, it is evident that gestational exposure to THC assists dysregulation of DA synthesis which is proficient in potential public health implications. However, the reports from animal and human experiments on cannabis exposure exhibited conflict on the results. It is found that acute THC elevates DA release and neuronal activity, while long-term exposure to THC promotes a blunted DA system. Alternatively, CBD is found to ameliorate the psychotic symptoms induced by THC and has been reported to maintain DAergic sustenance.

However, behavioral consequences of THC and CBD along with the knowledge on their persistence in DAergic effects, whether long-term or short-term is not well established. Therefore, it can be suggested for supplementary research on the long-term and developmental DAergic effects of cannabis. Moreover, the pragmatic effects of CBD are not clearly justified on THC-induced psychosis and DA synthesis. Much research is still anticipated on these findings and effects of other cannabinoids on behavioral anomalies accompanying both physical alterations and brain DA systems.

## Abbreviations

AMPH: Amphetamine
AST: Associative striatum
CBD: Cannabidiol
CB1R and CB2R: Endocannabinoid Receptor 1 and 2
DA: Dopamine
DOPAC: 3,4-Dihydroxy-phenylacetic acid
GABA: Gamma-aminobutyric acid
LST: Limbic striatum
mTOR: Mammalian target of rapamycin
MRS: Magnetic resonance spectroscopy
NAc: Nucleus accumbens
PET: Positron emission tomography
PFC: Prefrontal cortex
SMST: Sensorimotor striatum
THC: Tetrahydrocannabinol
VTA: Ventral tegmental area
